# Dissociable electrophysiological measures of natural language processing reveal differences in speech comprehension strategy in healthy ageing

**DOI:** 10.1038/s41598-021-84597-9

**Published:** 2021-03-02

**Authors:** Michael P. Broderick, Giovanni M. Di Liberto, Andrew J. Anderson, Adrià Rofes, Edmund C. Lalor

**Affiliations:** 1 School of Engineering, Trinity Centre for Bioengineering and Trinity College Institute of Neuroscience, Trinity College Dublin, Dublin 2, Ireland; 2 Laboratoire des Systèmes Perceptifs, Département d’études Cognitives, École Normale Supérieure, PSL University, CNRS, 75005 Paris, France; 3 Department of Biomedical Engineering, University of Rochester, Rochester, NY 14627 USA; 4 Department of Neuroscience, and Del Monte Institute for Neuroscience, University of Rochester, Rochester, NY 14627 USA; 5 Department of Neurolinguistics and Language Development, University of Groningen, Oude Kijk in Het Jatstraat 26, 9712 EK Groningen, The Netherlands

**Keywords:** Cognitive ageing, Cognitive neuroscience

## Abstract

Healthy ageing leads to changes in the brain that impact upon sensory and cognitive processing. It is not fully clear how these changes affect the processing of everyday spoken language. Prediction is thought to play an important role in language comprehension, where information about upcoming words is pre-activated across multiple representational levels. However, evidence from electrophysiology suggests differences in how older and younger adults use context-based predictions, particularly at the level of semantic representation. We investigate these differences during natural speech comprehension by presenting older and younger subjects with continuous, narrative speech while recording their electroencephalogram. We use time-lagged linear regression to test how distinct computational measures of (1) semantic dissimilarity and (2) lexical surprisal are processed in the brains of both groups. Our results reveal dissociable neural correlates of these two measures that suggest differences in how younger and older adults successfully comprehend speech. Specifically, our results suggest that, while younger and older subjects both employ context-based lexical predictions, older subjects are significantly less likely to pre-activate the semantic features relating to upcoming words. Furthermore, across our group of older adults, we show that the weaker the neural signature of this semantic pre-activation mechanism, the lower a subject’s semantic verbal fluency score. We interpret these findings as prediction playing a generally reduced role at a semantic level in the brains of older listeners during speech comprehension and that these changes may be part of an overall strategy to successfully comprehend speech with reduced cognitive resources.

## Introduction

Healthy ageing is accompanied by a myriad of sensory and cognitive changes. This includes a decline in working memory^1^ and episodic memory^2^ as well as hearing loss^3^ and a slowing in processing across cognitive domains^4^. Given that spoken language comprehension is a multifaceted cognitive skill involving all these processes, it is remarkable that it remains relatively stable across a healthy adult’s lifespan. An interesting question, therefore, is whether the neural systems supporting successful language comprehension undergo a strategic shift with age to maintain preservation in the face of decline^5–7^, resulting in measurable differences between younger and older adults engaged in comprehension tasks. Furthermore, a related question is whether such differences play into the reported extra difficulties that older adults experience in trying to follow everyday conversational speech, especially in challenging listening environments^8–10^. Electrophysiology studies have indicated that age-related differences exist in the neural signatures relating higher level linguistic processing^11^. This has been shown consistently in studies examining the N400 component.

The N400 component of the event-related potential (ERP) is most widely studied in relation to language processing^12,13^. It is characterised by a centroparietal negativity that is elicited 200–600 ms after word-onset and is strongest for words that are incongruent with their preceding context (e.g., “I take my coffee with cream and socks”). Several contrasting theories have been advanced to account for the N400. These include suggestions that the N400 reflects analysis of the low-level (e.g., orthographic or phonological) attributes of the unexpected (read or heard) word before that word is actually recognized^14^; that it represents the process of accessing the semantic meaning of the word^15^; or that it represents the process of incorporating the meaning of the word into its preceding context^16^. One idea that has the potential to unify several of these competing theories is that the N400 reflects the stimulus induced change in a multimodal neural network, wherein an implicit and probabilistic representation of sentence meaning is held^12,17^. Importantly, the state of this internal network can be shaped by predictions, such that information can be partially or fully activated before the arrival of bottom-up input^18^. This idea relies on the suggestion that listeners process speech predictively. In particular, it has been suggested that listeners use their internal representation of context to predictively pre-activate information at multiple representational levels during language comprehension^19^. This includes the processing of semantic categories^18,20–23^, event structure^18,24–26^, syntactic structure^24,25,27–32^, phonological information^33,34^ and orthographic information^34,35^. In addition at a *lexical* surface level, this could include the activation of representations of word identity^34,36^, whereas a *semantic* level relates to the activation of an upcoming word’s semantic features^23,37^. It is believed that this pre-activation occurs in parallel across these distinct representational levels as part of an interactive network with reciprocal connections between each level^19,38,39^.

Previous cognitive electrophysiology studies have consistently shown age-related differences in the amplitude and latency of the N400 component, indicating that changes do indeed occur in how older adults use context to facilitate the processing of words^40–46^. An important study by Federmeier and colleagues found that, for younger adults, N400 responses showed a graded facilitation for words depending on their semantic relationship with some predicted target. For example the sentence “I take my coffee with cream and …”, showed the largest N400 reduction for expected completions (sugar), but also showed reductions for unexpected but semantically related words (salt) compared to unexpected and unrelated words (socks)^23,47^. This was taken as strong evidence that the N400 component reflects, in part, the predictive preactivation of semantic features for upcoming words^37^. Crucially, older adults, as a group, showed weaker facilitation effects for unexpected but semantically related target words, although a cohort of older adults with higher verbal fluency scores showed more younger-like responses^47^.

Several related N400 studies have similarly concluded that prediction plays an overall reduced role in language comprehension for older adults^11^. This strikes somewhat of a discord with findings from cognitive audiology^48^, behaviour^49–51^ and eye-tracking^52–54^ literatures that have, in many cases, shown the preservation of prediction mechanisms or a higher reliance on context in the brains of older adults (recently reviewed by Payne and Silcox^55^). However, as mentioned above, it is possible that the N400 reflects contributions from parallel predictive processing at multiple linguistic levels. Therefore, the notion that the preactivation of information at distinct levels is differentially affected by ageing has the potential to reconcile these apparently contrasting literatures.

To explore this possibility, we leveraged a recent experimental framework to isolate neural correlates of prediction from these different levels in younger and older adults using natural, continuous speech and modern context-based language modelling^56,57^. This approach includes the variations in predictability at different levels that come with natural speech and allows for the derivation of interpretable neural correlates for different aspects of predictive language processing according to the language models used in analysing the neural data^58–60^. Furthermore, the use of natural speech material (e.g., listening to a story vs an experimental paradigm with multiple sentences/questions) adds to the ecological validity of observed effects and is less taxing on the attention of participating subjects than experiments involving artificially constructed sentences^61^. This seems important for reducing the potential confound of different levels of attentional engagement between older and younger subjects.

Given the differences in how the language models operate, we hypothesized that they could dissociate predictive processes at lexical and semantic representational levels in terms of how they contribute to the N400. Additionally, based on previous N400 literature^47^, we hypothesized that older participants would show a specific detrimental effect in their ability to preactivate semantic features of upcoming words, and that this effect would correlate with behavioural measures of verbal fluency—for example, the number of word of words they could produce belonging to a specific category in fixed amount of time.

## Results

**Figure 1:**
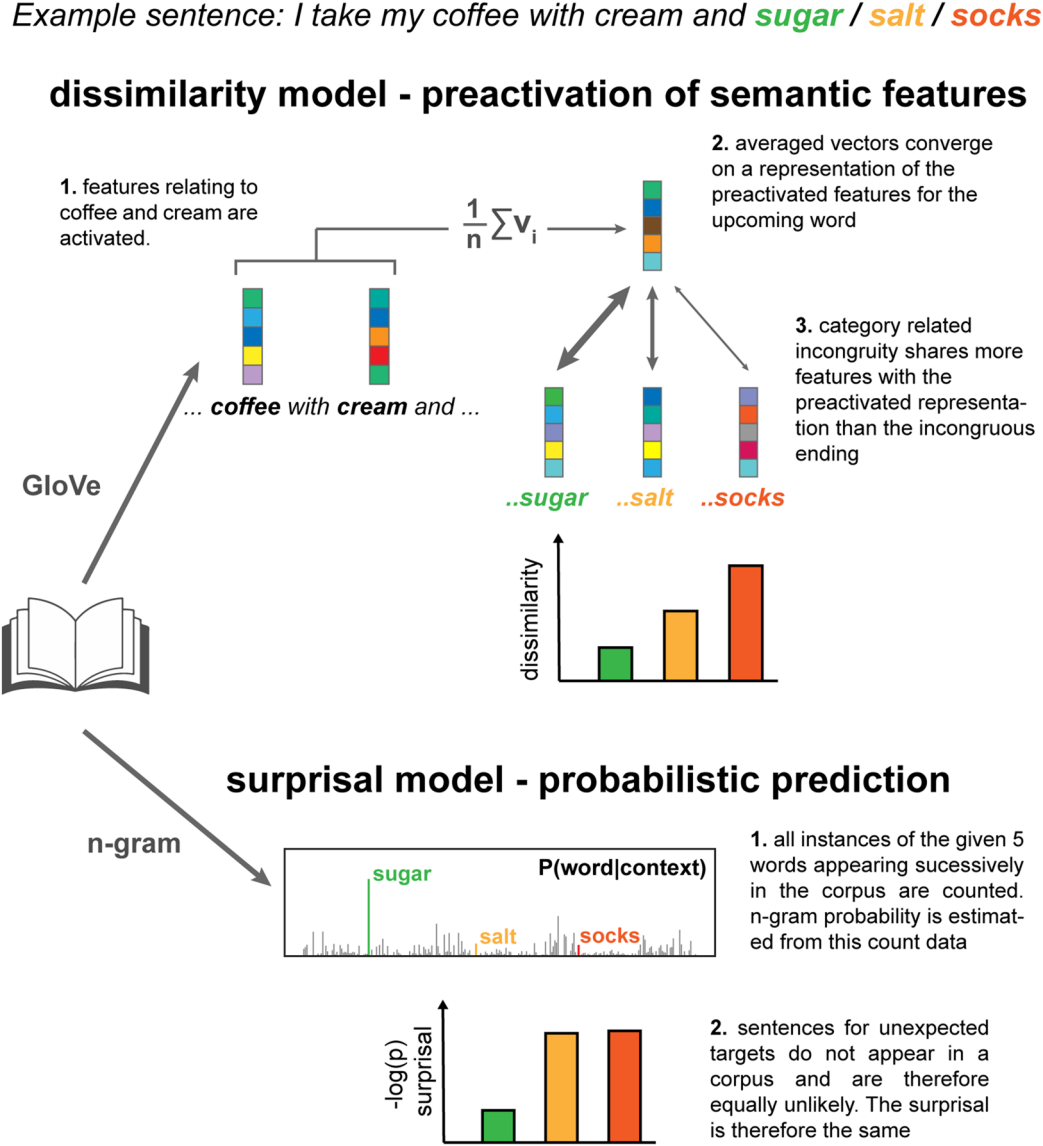
Computational models of predictive processing at lexical and semantic level. To illustrate the idea of prediction operating across multiple representational levels, consider the sentence *I take my coffee with cream and…* which ends with either an expected completion (*sugar),* an unexpected but semantically related completion (*salt*) or an unexpected and semantically unrelated completion (*socks*). At a lexical level, *salt* is unexpected because it is extremely rare that this sequence of words is heard or read. Processing of this word is therefore assumed to be no different from the processing of other unexpected words (i.e. *socks*). Conversely, at a higher *semantic* level, *salt* is relatively more likely, because *sugar* and *salt* share common features, both being powders and condiments; edible; white etc. We used two models of lexical surprisal and semantic dissimilarity to disentangle the contributions of prediction at lexical and semantic levels, respectively. Top: For the semantic dissimilarity model, vector representations of previous words in the sentence are averaged to form an estimation of the event context. The latent semantic features of the averaged vector converge on a representation similar to the predicted target “sugar” which, consequently, is more similar to words from the same category (e.g. “salt”) than different categories (e.g. “socks”). Bottom: Conversely, the lexical surprisal model does not distinguish between unexpected words based on their semantic category as it only reflects the probability of encountering either sequence of words in the training corpus, which is either rare or non-existent.

Two groups of 19 older (55–77 years, mean = 63.9) and 19 younger (19–38 years, mean = 26.8) subjects listened to the same 12-min long excerpt of narrative speech while their electrophysiological (EEG) signal was recorded. We first characterised the speech stimulus in terms of the predictive strength between each content and its preceding context at distinct linguistic levels. This was done using a recent modelling framework (Fig. 1) to tease apart neural correlates of predictive processing at the lexical and semantic level.

To model predictive processing at the lexical level, we estimated 5-g surprisal: an information theoretic measure of the inverse of the probability of encountering a word, given the ordered sequence of the 4 preceding words^62^. High lexical surprisal values arise from improbable word sequences. The surprisal estimate itself captures nothing about what individual words mean, in the sense that it supplies no measure of whether *cats* and *tigers* are categorically similar, or whether either are domestic. However, word symbol sequences do in part reflect the structure of meaningful events in the real world (*cats* chase *mice*). They also reflect grammatical constraints on permissible symbol sequences (“*The jumped the a cat”* is nonsensical). Lexical surprisal is highly correlated with cloze probability and can predict word reading times^63,64^.

To model predictive processing at the semantic level, we exploited a popular distributional semantic modelling approach that approximates word meaning, using numeric vectors of values reflecting how often each word co-occurred with other words across a large body of text^65^. Distributional modelling approaches like this support the construction of conceptual knowledge hierarchies, e.g., a dragonfly is an insect is an animal^66^, and would be expected to capture similarities between words belonging to similar categories, such as sugar and salt, and their difference to, say, socks. In addition they have been used to model N400 effects of semantic preactivation^23,67^ and have been shown to have a top-down influence on the early auditory encoding speech^68^. Semantic dissimilarity between a word and its preceding context was computed by 1 minus the Pearson’s correlation between the current word vector and the averaged vectors of all previous content words in the same sentence^57^.

In contrast to lexical surprisal, the semantic dissimilarity measure captures differences in the semantic categories that words belong to, and in the contexts that words appear in (*cats* and *tigers* are both felines, but *tigers* rarely occur in domestic contexts). Thus, semantic dissimilarity would predict a greater N400 for “I take my coffee with cream and *socks*”, than for “I take my coffee with cream and *salt”*^67^. In contrast, a 5-g surprisal model would likely regard *salt* and *socks* as being equally unexpected at the lexical level because their occurrences are both, presumably, non-existent in a text corpus. So, using this model, one might not expect to see much difference in the N400 for *salt* vs *socks*. (Fig. 1). We found that lexical surprisal and semantic dissimilarity were only weakly correlated (Pearson’s R = 0.14, p = 1.2 × 10^–5^, n = 916, Fig. S1A), indicating that they captured distinct features of the stimulus. Figure S1B provides example sentences from the experimental stimulus with lexical surprisal and semantic dissimilarity values of the final word.

The neural tracking of these speech features was assessed using a time-lagged linear regression. Specifically, this method models neural responses to speech by estimating a temporal filter that optimally describes how the brain transforms a speech feature of interest into the corresponding recorded neural signal. The filter, known as the temporal response function (TRF), consists of learned weights at each recorded channel for a series of specified time-lags. The TRF has typically been used to measure the cortical tracking of acoustic and linguistic properties continuous speech^69–71^. However, recent approaches using this method have sought to represent continuous speech beyond its low-level acoustic features, in terms of its higher-level lexical-semantic properties^57,68,72^.

**Figure 2:**
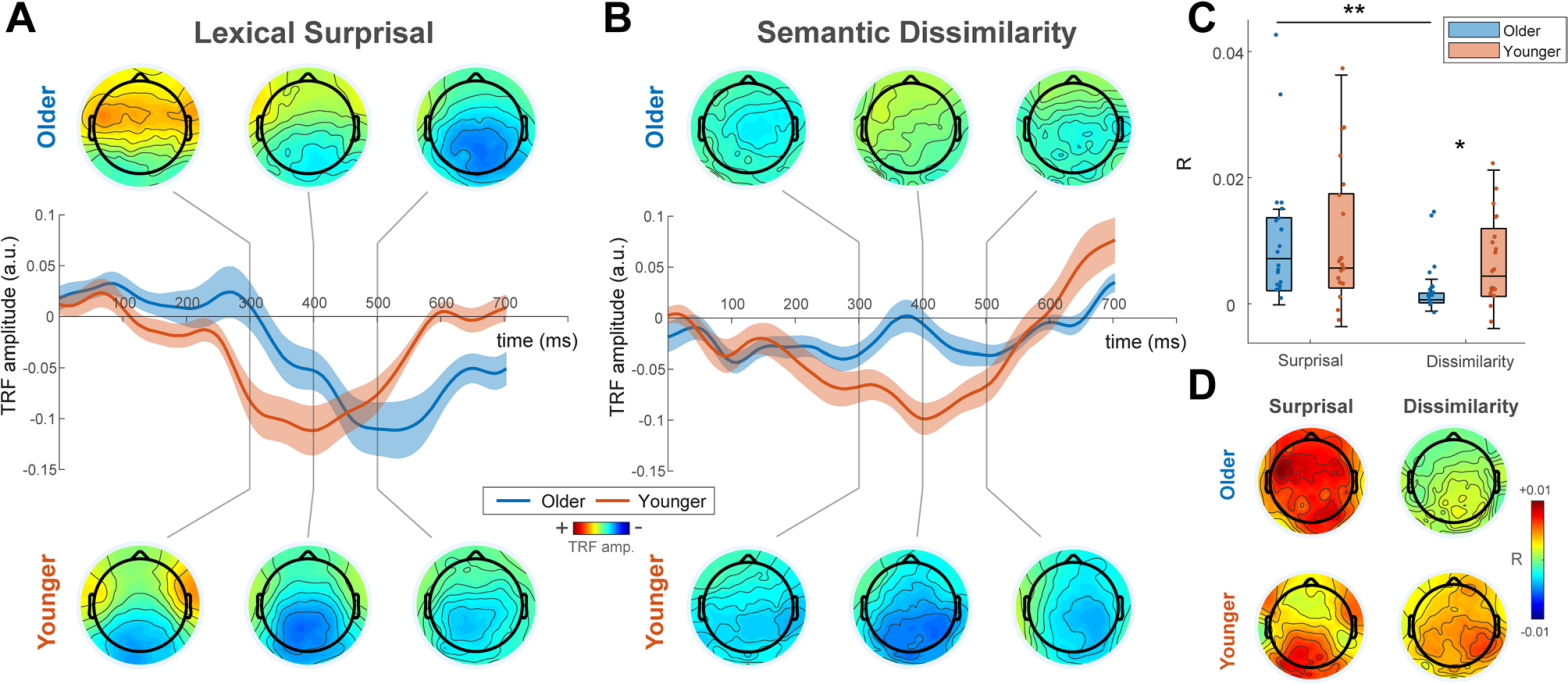
TRF weights. (**A**) Lexical surprisal TRF weights averaged over parietal electrodes and across older (blue) and younger (red) subjects. Shaded areas show s.e.m. across subjects. Scalp weight topographies at selected time windows (300 ms, 400 ms and 500 ms; window width of 50 ms) plotted above and below the channel plots for older and younger groups, respectively. N400 components were seen in the TRF weights at later time-lags for both groups and the peak latency of this component was significantly delayed for the older group. (**B**) Semantic dissimilarity TRF weights averaged over parietal electrodes and across older (blue) and younger (red) subjects. Shaded areas show s.e.m. across subjects. Scalp weight topographies at selected time windows (300 ms, 400 ms and 500 ms; window width of 50 ms) plotted above and below the channel plots for older and younger groups, respectively. In contrast to lexical surprisal, semantic dissimilarity TRF weights were significantly weaker at later time lags for the older group compared to the younger group. (**C**) EEG Prediction Accuracy. Boxplots of EEG prediction accuracy corresponding to each linguistic feature (lexical surprisal and semantic dissimilarity) and age-group. Dots indicate individual subjects. Consistent with the feature weights of the TRF, there were no significant differences between the prediction accuracies for dissimilarity and surprisal over parietal channels for younger subjects. However, older subjects showed significantly higher prediction accuracy for surprisal compared to dissimilarity at these channels. (**D**) Topographical plots of prediction accuracy for both age groups and both models.

To fit the TRF, lexical surprisal and semantic dissimilarity were represented as vectors of impulses at the onset of each content word whose heights were scaled according to their surprisal or dissimilarity value. We regressed these vectors simultaneously to the recorded EEG signal of each individual participant. This produced separate TRF weights for surprisal and dissimilarity. Figure 2A shows the surprisal TRF weights for older and younger groups at midline parietal electrodes with scalp weight topographies at selected time windows (300 ms, 400 ms and 500 ms; window width of 50 ms) plotted above and below. Both groups show a prominent negative component, characteristic of the classic N400 ERP. We found that the latency of this component was significantly delayed by 74 ms in the older group (T = 3.5, p < 0.005, 2 sample t-test, Cohen’s d = 1.13) and observed a correlation between age and response peak latency within the older group (Pearson’s r = 0.46, p = 0.047). Figure 2B shows the TRF weights for the semantic dissimilarity feature in older and younger subjects. Younger subjects showed comparable responses for dissimilarity and surprisal feature weights. In contrast, dissimilarity weights were significantly weaker than surprisal weights for older subjects (p < 0.05 running paired t-test, FDR corrected).

The performance of a model is also assessed by its ability to predict unseen neural data. Employing a cross validation procedure, we trained TRFs on a subset of each subject’s EEG data. This trained model was used to predict EEG that was compared with the remaining, held out EEG data. To test the predictive strength of surprisal and dissimilarity individually, we compared prediction accuracy of the full model (including dissimilarity and surprisal) with 5 null models where either surprisal or dissimilarity values were randomly permuted. Figure 2C shows the prediction accuracy (r) of each feature relative to the average null model predictions over midline parietal channels for younger and older subjects. Figure 2D shows the topographical distribution of r values. For both groups, surprisal and dissimilarity could significantly predict EEG above this baseline (Younger subjects: p = 0.0005 and p = 0.0011 for dissimilarity and surprisal, respectively; Wilcoxon signed-rank test. Older subjects: p = 0.022 and p = 0.0002, for dissimilarity and surprisal, respectively; Wilcoxon signed-rank test). Consistent with the feature weights of the TRF, there were no significant differences between the prediction accuracies for dissimilarity and surprisal for younger subjects (p = 0.28, Wilcoxon signed-rank test). However, importantly, older subjects showed significantly higher prediction accuracy for surprisal compared to dissimilarity (p = 0.0048, Wilcoxon signed-rank test). Younger subjects also showed significantly higher prediction accuracy for dissimilarity than older subjects (p = 0.033, Mann–Whitney U-test), whereas no significant age-related difference was found for lexical surprisal prediction accuracies (p = 0.91, Mann–Whitney U-test). 2 of the 19 subjects scored below 25 (scores of 24 and 23) in a prior screening Montreal Cognitive Assessment (MOCA). Although 25 is typically set as the inclusion criteria^73^, we chose to include these subjects in the main analysis because the MOCA is not a direct measure of language function. However, excluding these subjects did not affect the within group difference of semantic dissimilarity and lexical surprisal models in older adults (p = 0.015, Wilcoxon signed-rank test) or the between group difference for semantic dissimilarity (p = 0.043, Mann–Whitney U-test).

From these results it is evident that semantic dissimilarity is weaker at explaining the neural responses for older subjects compared to younger subjects. However, this difference in model performance could conceivably be due to the particular way in which we have computed semantic dissimilarity. For instance, it has been shown that older adults have reduced working memory capacity^1^, and thus for older adults it may be more appropriate to compute dissimilarity using a smaller window of previous words. To safeguard against this possibility, we tested several semantic dissimilarity vectors, where dissimilarity was estimated by comparing a word with a fixed number of previous words. We used context window sizes of 3, 5, 7, 9 and 11 words. We found no differences between models with different context window sizes or the model with a sentence context window (p = 0.61 for the older group, p = 0.79, for the younger group, Kruskal–Wallis test)*,* indicating that the difference in brain responses between younger and older participants was not the result of the selected parameters.

Finally, we investigated the low-level acoustic tracking of the speech envelope in both groups to check if our results might be explained based on differences in processes impacting in low-level encoding of the speech signal. Envelope tracking is sensitive to factors like attention or perceived speech intelligibility^74–77^ and weaker tracking measures in the older group could indicate that these processes negatively impact subjects’ ability to preactivate semantic features of upcoming words. Consistent with previous reports^9,78^, we found significantly *stronger* tracking of the speech envelope in older adults (Fig. S2), suggesting that low-level acoustic processing does not explain the between-group differences we see in semantic dissimilarity.

**Figure 3:**
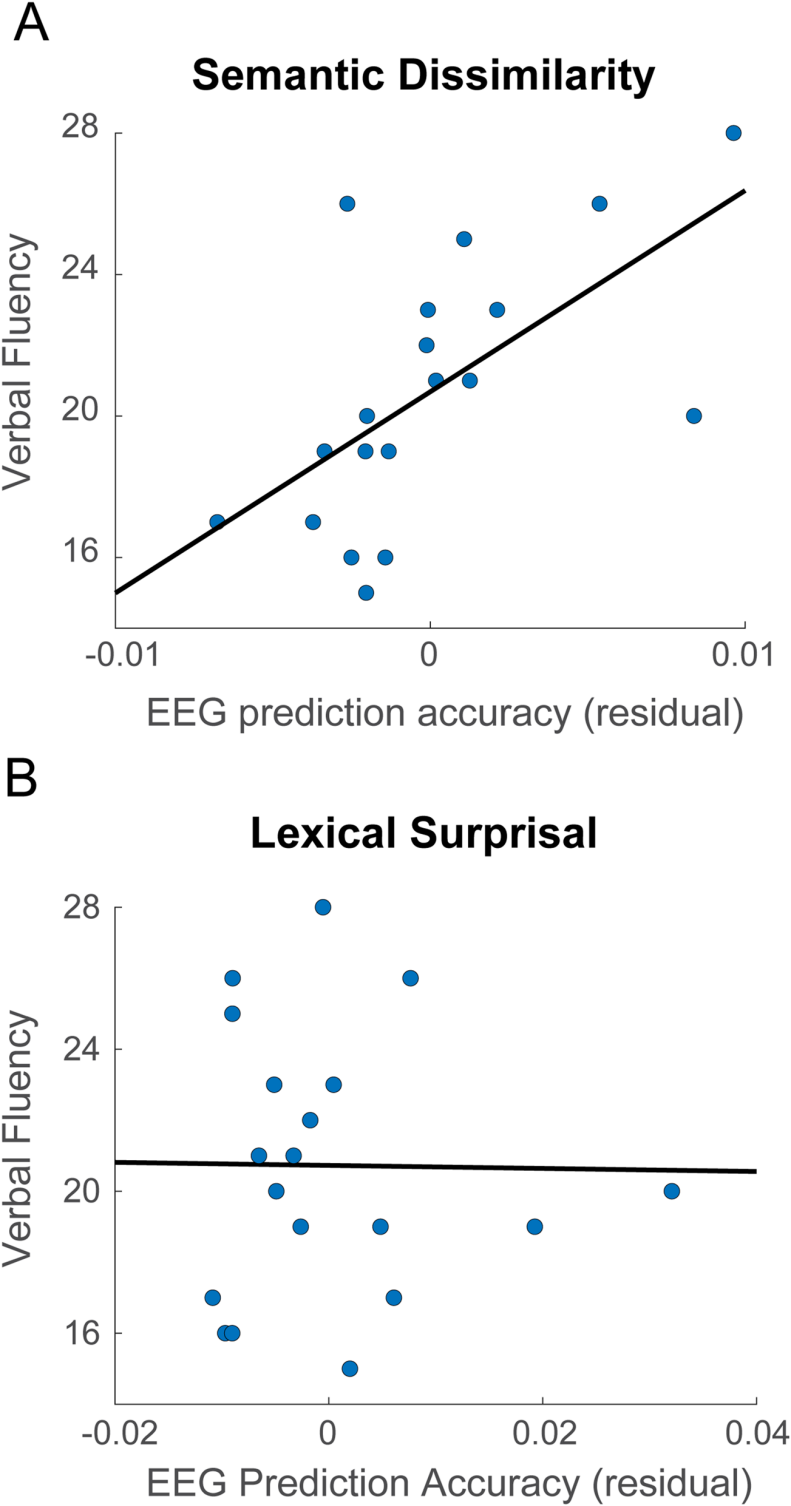
(**A**) Within the older subjects, semantic category fluency was positively correlated with the semantic dissimilarity model performance when controlling for age (R = 0.63 p = 0.005). (**B**) In contrast, semantic category fluency was uncorrelated with the lexical surprisal model performance when controlling for age (R = − 0.04 p = 0.88).

Previous work has indicated that older adults with higher verbal fluency scores were more likely to engage predictive processes at the level of semantics, resulting in N400 response patterns that were more similar to their younger counterparts^47^. On this basis, we tested whether semantic dissimilarity model performance could predict verbal fluency in our older subjects. We found that, when controlling for age, model prediction accuracies were positively correlated with semantic fluency scores (i.e. the total number of words produced from a semantic category in 60 s) across subjects (Pearson’s R = 0.63, p = 0.005, Fig. 3). This reveals that semantic dissimilarity was more accurately modelled for older subjects with higher semantic fluency. The model accuracy for surprisal was not predictive of this measure (Pearson’s R = − 0.04, p = 0.88). In addition, model accuracies for surprisal and dissimilarity were not significantly correlated with letter fluency scores (R = − 0.12, p = 0.67; semantic dissimilarity, R = − 0.44, p = 0.07; lexical surprisal). Although the sample of the correlation was relatively low (n = 19) and verbal fluency measures are typically more robust when more than one construct is assessed per measurement^79^, this significant correlation represents a positive advance in the ability to acquire cognitive estimates from an individual’s neural signal under naturalistic listening conditions.

## Discussion

The current article has revealed differences between younger and older adults’ electrophysiological responses to natural, narrative speech. In both young and old, a joint model capturing lexical surprisal and semantic dissimilarity produced N400 component responses in its temporal weights. While the lexical surprisal measure was robust in older adults, its peak negativity was delayed, similar to previous reports based on the N400^43^. In contrast, the semantic dissimilarity measure was much reduced in the older subjects. We interpret this as evidence for two distinct contributions to the N400 that reflects how information is predictively preactivated at lexical and semantic levels of linguistic representation. Furthermore, the pattern of results suggests that while older subjects maintain a robust ability to utilize lexical predictions during language processing, their ability to do so based on semantic representations appears to be impaired. Importantly, this interpretation was supported by the fact that the performance of the semantic dissimilarity model in older adults reflected a semantic behavioural measure of their categorical verbal fluency. These results extend basic scientific understanding of neurophysiological changes that accompany ageing and could have implications for research into naturalistic measures of brain health, as we discuss below.

The notion that our measures reflect dissociable neural processes contributing to the N400 fits with results from previous modelling studies on younger adults. Surprisal and dissimilarity measures were jointly modelled on both EEG and fMRI responses in younger adults during sentence reading and narrative speech comprehension respectively^58^. The measures both produced similar N400 responses in the sentence reading EEG data, as they have done for the younger adults in our study (Fig. 2). However, the fMRI results provided evidence that distinct brain regions were involved in processing the two different aspects of the speech input. In particular, visual word form areas reflected surprisal (i.e., lexical processing) and areas of the semantic network^80^ reflected dissimilarity (i.e., semantic processing). In that study, the distinct contributions of lexical and semantic processing were not dissociable in the N400 data because they were both strongly represented in younger adults. Our study goes beyond this, in 1) revealing that electrophysiological activity elicited in narrative speech comprehension reflects components of lexical surprisal and semantic dissimilarity; 2) demonstrating an age-related dissociation between the contribution of surprisal and dissimilarity, with the dissimilarity component being less pronounced in older people. Together, these findings provide convergent evidence that the N400 response reflects contributions from multiple processes relating to prediction at different levels of the linguistic processing hierarchy^19,81^.

The idea that the N400 is affected by prediction at lexical and semantic levels also aligns well with previous N400 ERP literature. N400 ERP responses in younger adults were modulated for words that were not only unexpected in their context, but also belonged to a different semantic category to the expected word^23^. This is consistent with semantic features of upcoming words being predictively preactivated by a comprehender’s internal representation of context in parallel with the preactivation of word form at a lexical level. Importantly, the relative contribution of lexical and semantic components to the N400 appears to change with age. Specifically, in older adults there is less sensitivity to an unexpected word's semantic category, especially when the preceding context is highly constraining. These results are consistent with the idea of prediction playing a reduced role at a semantic level in the ageing brain^41,47^. However, whereas N400 ERP paradigms propose that different sub processes contribute to the N400 based on how evoked responses vary as a function of sentence-ending, our approach has dissociated lexical surprisal and semantic dissimilarity in neural responses to a continuous stretch of narrative speech.

We show that prediction of upcoming lexical items is preserved with age. This was captured using a Markov model. Although Markov (lexical surprisal) models perform well at next word prediction tasks despite being a lot simpler than artificial neural networks, they suffer from an inability to generalize^82^. For example, they are insensitive to the fact that sentences like *I take my coffee with cream and sugar* and *I like my espresso milky and sweet* mean similar things. Thus, they represent a more rigid probabilistic model of language that still works quite well. This model could correspond to a design feature of a lexical system, specialised to use meaningful context to predict upcoming word identity rather than more abstract linguistic information, such as a word’s semantic features. This lexical system operates similarly in younger and older adults whose accumulated knowledge of language is more crystalized; less fluid^50^ and reflects the same rigidity as the Markov model. In contrast, ageing results in a lesser engagement in a semantic system that is specialised in the prediction of semantic information.

Hence, our modelling approach suggests that predictive processing is preserved with age and that older adults draw on their internal representation of context to facilitate the processing of incoming information. This aligns with findings from eye tracking, behaviour, and cognitive audiology literatures. However, the information that is preactivated represents a more rigid commitment to the identity of upcoming words. The preactivation of linguistic information that exists at semantic levels of representation, captured by semantic dissimilarity, that can generalize across words is much weaker in the brains of older listeners, although subjects with higher verbal fluency scores exhibited stronger preactivation. This could explain why word reading time context effects are preserved with age, as word reading times correlate with lexical surprisal^63^, and not with semantic dissimilarity when surprisal is factored out^58^. In contrast, both semantic dissimilarity and lexical surprisal can explain variance in N400 responses when controlling for one another^58^. Based on the notion that the N400 reflects contributions from multiple distinct linguistic levels, this could explain why weaker N400 responses are consistently observed in older adults^11,40,41,43^.

Older adults show remarkably preserved language comprehension skills despite experiencing an overall decline in sensory and cognitive function^83^. A lesser reliance on predictions at a semantic level may be part of a strategy to successfully comprehend speech with reduced cognitive resources^84^. This possibly highlights the putative value of high-level predictions to support speech comprehension in noisy environments, when the input is corrupted and where older adults often struggle to comprehend. Future work, presenting speech at different levels of signal-to-noise ratio could help our understanding of such phenomena.

We wish to further emphasize that the study was undertaken using a short, 12-min segment of natural continuous speech stimulus. Previous research into the electrophysiological changes in language processing in the ageing brain have leveraged ERP-based experimental protocols that rely on experimenter-configured stimulus sets to enable contrasts between different stimulus conditions (e.g. congruent and incongruent sentence wordings). However, the ERP approach constrains the breadth of linguistic stimuli that can be investigated to the subset of sentences configured into matched experimental pairs. Additionally, the degree of ecological validity of results generated from bespoke ERP setups is unclear because the experimental conditions are rarely experienced in everyday life. By examining electrophysiological responses elicited in audiobook comprehension, we have utilized a stimulus that is actually experienced in the wild, and the participant experiences an uninterrupted prolonged and cohesive discourse, that is likely to be more engaging than listening to disjoint experimental sentences.

In conclusion, we have revealed neural correlates of language prediction relating to distinct measures of lexical surprisal and semantic dissimilarity. We show how one of these forms of prediction becomes less effective with age and patterns with behavioural cognitive measures, enabling us to derive accurate estimates an individual’s verbal semantic fluency from their neural data alone. These findings open new possibilities to study language impairment in the elderly and detect the onset of neurodegenerative disorders.

## Materials and methods

### Participants

Data from 38 individuals (19 younger (6 female), age 19–38 years, M = 26.8 years ± s.d. = 5 years; 19 older (12 female), age 55–77 years, M = 63.9 years ± s.d. = 6.7 years) were reanalysed for this study. Data from the 19 younger subjects was collected in previous studies^57,71^ and in the current analysis only a portion of each subjects’ data was used (the first 12 min) in order to match the amount of recorded data for the older participants. Data collected from the 19 older subjects had not been previously published. Both studies were undertaken in accordance with the Declaration of Helsinki and were approved by the Ethics Committee of the School of Psychology at Trinity College Dublin. Each subject provided written informed consent. Subjects reported no history of hearing impairment or neurological disorder.

### Stimuli and experimental procedure

The stimulus was an audio-book version of a popular mid-twentieth century American work of fiction (The Old Man and the Sea, Hemingway, 1952), read by a single male American speaker. The first 12 min of the audiobook was divided into 4 trials, each 3 min in duration. The average speech rate was 190 words/minute. The mean length of each content word was 334 ms with standard deviation of 140 ms. Trials were presented chronologically to the story with no repeated trials. All stimuli were presented monophonically at a sampling rate of 44.1 kHz using Sennheiser HD650 headphones and Presentation software from Neurobehavioural Systems. Testing was carried out in a dark, sound attenuated room and subjects were instructed to maintain visual fixation on a crosshair centred on the screen for the duration of each trial, and to minimise eye blinking and all other motor activities. Procedural instructions did not differ between younger and older subject groups. Prior to the experiment, subjects were not questioned about their familiarity with the story, however no subject reported being overly familiar with the story upon hearing it.

Older participants were additionally tested with 2 verbal fluency (VF) tasks. Letter verbal fluency was measured by asking participants to name as many words beginning with the letter ‘F’ as they could in 60 s^85^. Similarly, semantic verbal fluency was measured by asking participants to name as many animals as they could in 60 s^86^. These measures have been shown as reliable indicators of verbal fluency in older adults^87,88^. Audio recordings of participant responses were acquired and transcribed verbatim. Average subject letter fluency was 14.63 ± 4.27 S.D. Average subject category fluency score was 20.7 ± 3.75 S.D.

Prior to the verbal fluency task, participants were screened using the Montreal Cognitive Assessment (MOCA). Mean MOCA score was 26.7 ± 1.9 S.D. Verbal fluency is usually assessed as part of MOCA using the letter fluency task. A point is added to the overall MOCA score if a subject correctly names 11 or more words beginning with ‘F’. To avoid potential repetition effects, subjects performed the letter fluency task only once and both MOCA and letter fluency scores were obtained from this task.

### EEG acquisition and preprocessing

128-channel EEG data were acquired at a rate of 512 Hz using an ActiveTwo system (BioSemi). Offline, the data were downsampled to 128 Hz and bandpass filtered between 0.5 and 8 Hz using a zero-phase shift Butterworth 4^th^ order filter. Previous studies investigating the cortical tracking of continuous speech have used similar cut-off frequencies when filtering the EEG signal^57,71,76^. To identify channels with excessive noise, the standard deviation of the time series of each channel was compared with that of the surrounding channels. For each trial, a channel was identified as noisy if its standard deviation was more than 2.5 times the mean standard deviation of all other channels or less than the mean standard deviation of all other channels divided by 2.5. Channels contaminated by noise were recalculated by spline interpolating the surrounding clean channels. Data were then referenced to the average of the 2 mastoid channels.

Finally, we applied multiway canonical component analysis (MCCA) to denoise the data. MCCA is a technique that seeks to extract canonical components across subjects^89^. Like CCA, which is applied to single subjects, it can be used to find linear components that are correlated between stimulus and response. However, rather than analysing the components directly, EEG can be denoised by projecting it to the overcomplete basis of canonical components, selecting a set of components and then projecting back to EEG space. We denoised each age-group separately, with the prior hypothesis that latency and morphology of the group responses would be different. For each group, we chose parameters of 40 principal components for the initial principal component analysis and then 110 canonical components. These chosen values were based on the parameters that were recommended for denoising speech related EEG^89^; however, we tried several different parameter pairs and tested their effect on the prediction accuracy of EEG from the speech envelope. We found that the recommended parameters returned the optimal denoising for both groups as determined by prediction accuracy of EEG based on the speech envelope (Figure S2).

### Semantic dissimilarity and surprisal estimation

#### Semantic dissimilarity

Distributed word embeddings were derived using GloVe^65^. This method factorizes the word co-occurrence matrix of a large text corpus, in this case Common Crawl (https://commoncrawl.org/). The output is 300-dimensional vectors for each word, where each dimension can be thought to reflect some latent linguistic context. These word embeddings are used to calculate our semantic dissimilarity measure. This is an impulse vector, the same length as a presented trial, with impulses at the onset of each content word. The height of each impulse is 1 minus the Pearson’s correlation between that word’s vector and average of all preceding word vectors in the same sentence. Semantic dissimilarity values have a mean of 0.48 ± s.d. = 0.17.

#### Surprisal

Surprisal values were calculated using a Markov model trained on the same corpus as GloVe (common crawl). These models, commonly referred to as n-grams, estimate the conditional probability of the next word in a sequence given the previous *n-1* words. We applied a 5-g model that was produced using interpolated modified Kneser–Ney smoothing^62,90^. Surprisal vectors were calculated as impulses at the onset of all words whose heights were scaled according to the negative log of a word’s 5-g probability. Like the semantic dissimilarity vector, impulses for any non-content words were removed resulting in the removal of any impulses that were not common between dissimilarity and surprisal vectors. Surprisal values were normalised to match the distribution of dissimilarity values with a mean of 0.48 ± s.d. = 0.16.

### Temporal response function

The forward encoding model or temporal response function (TRF) can be thought of as a filter that describes the brain’s linear mapping between continuous speech features, S(t), and continuous neural response, R(t).

$$ R\left( t \right) = TRF*S\left( t \right) $$

where ‘*’ represents the convolution operator. The speech input can comprise of a single speech feature, i.e. univariate, or multiple speech features, i.e. multivariate. Each feature produces a set temporal weights for a series of specified time lags. TRF weights are estimated using ridge regression.

$$ TRF = \left( {S^{T} S + \lambda I} \right)^{ - 1} S^{T} r $$

where λ is the regularization parameter that controls for overfitting. The models are trained and tested using a fourfold cross-validation procedure. 3 of the 4 trials are used to train the TRF which predicts the EEG of the remaining trial, based on speech representation input. We train and test models based on the combined semantic dissimilarity and surprisal impulse vectors with the addition of an onset impulse vector with impulse height equal to the average dissimilarity and surprisal values across all words in the current trial. The onset vector acts as a nuisance regressor to capture variance relating to any acoustic onset responses. For testing, the prediction accuracy (R) of the model is calculated as the Pearson’s correlation between the predicted EEG and the actual EEG. A range of TRFs were constructed using different λ values between 0.1 and 1000. The λ value corresponding to the TRF that produced the highest EEG prediction accuracy, averaged across trials and channels, was selected as the regularisation parameter for all trials per subject. Previous analyses have linked similar regression-based methods with ERP components^60,91,92^, providing a methodological baseline for our current analysis. In particular, these methods can yield N400 responses using cloze probability^60,91,93^ and word frequency^94,95^. These studies are based on the same time-lagged linear regression approach as ours but typically analyse EEG responses to discrete events, such as individual words at the end of sentences. Our analysis extends this work to more natural, continuous stimuli. We do, of course, anticipate differences between the neural processing of discrete and continuous stimuli because, in the latter case, the context that accrues as the narrative progresses is coherent. Future work will seek to investigate these differences.

To test directly how well each feature accurately captured neural activity for each subject we measured the model’s ability to predict EEG based on the true feature representation above null feature representations. Specifically, the heights of the impulses for the semantic models were randomly shuffled to produce permuted dissimilarity or permuted surprisal vectors. In the testing phase of the cross-validation procedure, a trained TRF would attempt to predict the neural response to the permuted features, while all other features remained constant. This was repeated for 5 permutations of each stimulus feature. Hence, prediction accuracy for semantic features refers to the prediction accuracy difference between true speech feature and the average of the 5 null speech feature representations.

In addition, we extracted properties of the model weights themselves. N400 peak latency was calculated automatically for each subject as the time lag with the lowest peak weight within a window of 200–600 ms after a time-lag of zero. The response peak delay between groups was calculated as the difference between group averaged peak delays.

### Statistical testing

For every statistical comparison, we first verified whether the distribution of the data violated normality and was outlier free. This was determined using the Anderson–Darling test for normality and 1.5 IQR criterion, respectively. We used parametric tests (t-test, paired t-test) for data which satisfied these constraints and non-parametric tests for data which violated them.

## Supplementary Information

Supplementary Information

## Data Availability

Raw EEG data for the younger subjects is available to download from https://doi.org/10.5061/dryad.070jc. Raw EEG data for the older subjects is available upon request.
